# Neural encoding of the speech envelope by children with developmental dyslexia

**DOI:** 10.1016/j.bandl.2016.06.006

**Published:** 2016-09

**Authors:** Alan J. Power, Lincoln J. Colling, Natasha Mead, Lisa Barnes, Usha Goswami

**Affiliations:** Centre for Neuroscience in Education, University of Cambridge, Downing St, Cambridge CB2 3EB, UK

**Keywords:** Dyslexia, Oscillations, Phonology, Rhythm

## Abstract

Developmental dyslexia is consistently associated with difficulties in processing phonology (linguistic sound structure) across languages. One view is that dyslexia is characterised by a cognitive impairment in the “phonological representation” of word forms, which arises long before the child presents with a reading problem. Here we investigate a possible neural basis for developmental phonological impairments. We assess the neural quality of speech encoding in children with dyslexia by measuring the accuracy of low-frequency speech envelope encoding using EEG. We tested children with dyslexia and chronological age-matched (CA) and reading-level matched (RL) younger children. Participants listened to semantically-unpredictable sentences in a word report task. The sentences were noise-vocoded to increase reliance on envelope cues. Envelope reconstruction for envelopes between 0 and 10 Hz showed that the children with dyslexia had significantly poorer speech encoding in the 0–2 Hz band compared to both CA and RL controls. These data suggest that impaired neural encoding of low frequency speech envelopes, related to speech prosody, may underpin the phonological deficit that causes dyslexia across languages.

## Highlights

• We measure encoding quality of low-frequency speech envelopes by children using EEG.
• Encoding accuracy is significantly above chance for all groups.
• Accuracy is poorer in dyslexic children than younger RL-matched children.
• Individual differences in encoding accuracy are related to prosodic awareness.

## 1 Introduction

Children with developmental dyslexia have difficulty in processing the phonological aspects of speech, across languages (Ziegler & Goswami, 2005, for review). For example, they are poor at making decisions about whether words rhyme with each other (“cat” “hat”), at counting syllables in words (“caterpillar” has 4 syllables), at detecting syllable stress (“difficulty” has first syllable stress) and at deleting individual speech sounds (phonemes: “star” without the “s” sound leaves “tar”) (e.g., Bradley & Bryant, 1978, English; Wimmer, 1993, Wimmer, 1996, German; Share & Levin, 1999, Hebrew; Kim & Davis, 2004, Korean). These phonological difficulties are found not only when children with dyslexia are compared to chronological age-matched children without reading difficulties (the CA match design), but also when children with dyslexia are compared to younger children matched for reading level (the RL match, designed to equate the effects of reading experience on the brain; Goswami, 2003). Furthermore, training phonology improves reading acquisition for all children (e.g., Bradley and Bryant, 1983, Lundberg et al., 1988, Schneider et al., 1997), and also improves visual processing in dyslexia (Olulade et al., 2013). Accordingly, the phonological difficulties experienced by children with dyslexia are considered a causal factor in this developmental disorder (Goswami, 2015). Consequently, current remediation relies on intensive phonological training at the phoneme level accompanied by training in letter-sound correspondences (e.g., Brem et al., 2010, Schneider et al., 2000).

Accurate encoding of the phonological structure of words requires efficient auditory processing. Recent studies with adults and children with developmental dyslexia have consistently reported atypical neural activity related to auditory processing (Abrams et al., 2009, Lehongre et al., 2011, Poelmans et al., 2012, Hornickel and Kraus, 2013, Power et al., 2013, Lizarazu et al., 2015). However, none of these recent auditory studies has used a reading level (RL) match control group, an important research design for helping to distinguish cause from effect in studies of developmental disorders (Goswami, 2003). When children with dyslexia show impairments compared to *both* age-matched peers and to younger children matched for reading achievement, this suggests a causal role, as impairments occur despite matching for both developmental level and reading level. Intervention studies can then be used to investigate the causal status of identified factors. Accordingly, inclusion of an RL-matched control group may help to determine whether the observed differences in neural activity in recent auditory studies are a cause of dyslexia or a consequence of the atypical (severely reduced) reading experience that accompanies having dyslexia.

Some neural studies using developmental research designs are now beginning to emerge in the literature. These include longitudinal studies (Krafnick, Flowers, Luetje, Napoliello, & Eden, 2014), studies incorporating an RL-matched group to control for reading experience (Clark et al., 2014, Olulade et al., 2013), studies of pre-readers (Saygin et al., 2013) and studies including unaffected at-risk groups in an attempt to find endophenotypic traits (Khan et al., 2011, Leppänen et al., 2012, Neuhoff et al., 2012). For example, Saygin et al. (2013) studied pre-reading children with a range of phonological abilities. They found significant links between pre-reading phonological skills and the integrity of white matter organisation in the left arcuate fasciculus (Saygin et al., 2013). Krafnick et al. (2014) used an RL- matched control group and fMRI to show that previously-reported differences in grey matter volume between dyslexics and controls arise largely from the disordered reading experience that ensues from being dyslexic, rather than being causal to the disorder (Krafnick et al., 2014). In a longitudinal neuroanatomical study beginning with pre-reading children at-risk for dyslexia, abnormalities in the left-lateralised reading network were only observed *after the children had learned how to read* (Clark et al., 2014). In this small-scale study, the neuroanatomical *precursors* to dyslexia were restricted to the primary sensory cortices (Clark et al., 2014, Goswami, 2014). Meanwhile, an RL-match study exploring the role of visual sensory processing in dyslexia showed that abnormal visual motion processing was a result of impaired reading experience rather than a cause of dyslexia (Olulade et al., 2013).

These recent studies show the power of developmental research designs in distinguishing the causes and consequences of developmental dyslexia. In the electrophysiological (EEG) literature, however, developmental research designs are largely absent. For example, Schulte-Korne and Bruder (2010) reviewed over 30 EEG studies of sensory processing in children and adults with developmental dyslexia, yet none of the studies reviewed included an RL-matched control group to control for the effects of reading experience on the brain. Hämäläinen, Salminen, and Leppänen (2013) reviewed over 50 studies of non-speech auditory processing in developmental dyslexia, including 17 EEG studies. Again, none of the EEG studies reviewed included an RL-matched control group. Some EEG studies have employed an unaffected at-risk group in an attempt to control for reading experience. For example, Neuhoff et al. (2012) compared dyslexic children to unaffected age-matched siblings as well as to unaffected not-at-risk CA controls. The unaffected siblings had a genetic risk for dyslexia but had normal reading and spelling abilities. Neuhoff et al. reported that the late MMN to tone burst stimuli was diminished in both the dyslexic and the unaffected at-risk siblings compared to the CA controls. To our knowledge, the encoding of *connected speech* in developmental dyslexia has not yet been investigated electrophysiologically using an RL-matched group of younger children. Here, we investigate the encoding of sentences in children with developmental dyslexia using EEG and both CA- and RL-matched control groups. An RL control group is crucial in order to disambiguate the effects of reading experience on neural aspects of spoken language processing.

We explored the neural processing of slow temporal information in connected speech as a test of Temporal Sampling theory (Goswami, 2011). Temporal sampling theory predicts impaired neural encoding of speech envelope information in developmental dyslexia. We designed a novel test of temporal sampling theory using recent technical advances that enable speech resynthesis using EEG data (e.g., Mesgarani, David, Fritz, & Shamma, 2009). The resynthesis technique enables the speech stimulus to be reconstructed from the responses of the neuronal populations that encode it. A reverse reconstruction approach is used to find the best approximation of the input stimulus, and this best approximation is then compared to the original stimulus, for example via a linear mapping between features. The accuracy of the reconstruction is described as a correlation. Speech resynthesis techniques thus enable stimulus envelope reconstruction at the level of individual sentences and items (Mesgarani et al., 2009, O’Sullivan et al., 2014).

**Fig. 1:**
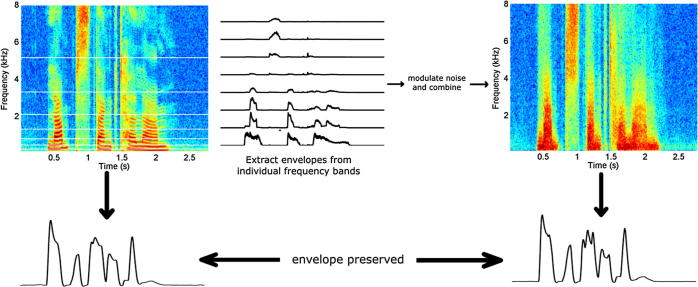
The noise-vocoding technique. The speech is filtered into a number of frequency bands. Within each frequency band the envelope is obtained by way of the Hilbert transform. This envelope is then used to modulate band pass filtered noise of the same bandwidth as the initial frequency band. These speech-amplitude modulated narrow-band noise signals are then recombined resulting in the noise-vocoded speech signal. Noise-vocoding degrades the spectral content of the speech. However, since the envelope is maintained throughout the noise-vocoding process the overall envelopes before and after vocoding are preserved. The number of frequency bands can be chosen at will. The lower the number of frequency bands used, the more degraded and unintelligible the speech is. Here we employed 8-channel vocoding to ensure some intelligibility while keeping performance from reaching ceiling.

Accordingly, by reconstructing individual speech stimulus envelopes from their resultant EEG patterns, a direct measurement of the neural encoding of speech by children becomes possible. This was our approach in the current study. We administered a word report task using noise vocoded speech that had been developed for children (Johnson, Pennington, Lowenstein, & Nittrouer, 2011), while simultaneously recording EEG. Noise vocoding degrades the temporal fine structure (TFS) of speech (see Fig. 1) while leaving the low frequency envelope intact. When the TFS of speech is degraded, listeners are forced to rely largely on the preserved envelope information in order to perceive the words and the sentences accurately. Although accurate listening is also supported by semantic information, here we deliberately used sentences that were semantically unpredictable (while being syntactically appropriate, e.g., “Arcs blew their cough”). Therefore, childrens’ ability to report the words and sentences accurately should enable assessment of the quality of their neural encoding of low frequency envelopes in speech. On temporal sampling theory, the quality of neural encoding for these low frequency envelopes should be impaired for children with dyslexia.

Utilising a developmental research design, we compared the neural encoding of low frequency speech envelopes by children with dyslexia with neural encoding by both CA-matched and RL-matched typically-developing control children. If children with dyslexia show significantly poorer speech encoding compared to younger children who can read the same number of words (the RL match design), the dyslexic deficit is less likely to arise from reduced reading experience (Goswami, 2003, Goswami, 2015). We assessed envelope reconstruction accuracy in 5 frequency bands (0–2, 2–4, 4–6, 6–8, 8–10 Hz). Following prior work, reconstruction accuracy was estimated by the Pearson correlation between the actual stimulus envelope of each sentence and the EEG reconstruction (Mesgarani et al., 2009, O’Sullivan et al., 2014). In speech resynthesis studies to date, reconstruction effects for neurotypical adults listening to connected speech (e.g., in a cocktail party paradigm) have yielded significant median Pearson correlations in the range of 0.05 (e.g., O’Sullivan et al., 2014).

All our sentences consisted of four monosyllabic words, hence had a relatively predictable temporal pattern. Recent research on temporal prediction has highlighted the relevance of delta-beta phase-amplitude cross-frequency coupling (Arnal, Doelling, & Poeppel, 2014). To explore the potential contribution of these temporal prediction networks to our sentence encoding task, we compared the topographies of delta-beta phase-amplitude coupling between our groups. Beta band activity has also been characterised recently as playing a privileged role in speech processing (see Poeppel, 2014). Accordingly, we also explored topographical differences in beta power between the children.

## 2 Materials and methods

### 2.1 Participants

Forty-six children participated in the study, who were all taking part in a longitudinal behavioural study of auditory processing (Goswami et al., 2013). Participants comprised all children in the cohort (of over 100 children) who volunteered for EEG: 12 children with dyslexia (DY), 23 typically-developing children matched for chronological age (CA), and 11 matched for reading level (RL, see Table 1 for detail). All participants and their guardians gave informed consent for EEG in accordance with the Declaration of Helsinki, and the study was approved by the Psychology Research Ethics Committee of the University of Cambridge. All participants were free of any diagnosed learning difficulties aside from dyslexia (i.e., dyspraxia, ADHD, autistic spectrum disorder, speech and language impairments) and spoke English as their first language.

### 2.2 Standardized tests of reading, nonword reading, vocabulary and IQ

Psychometric tests were given to reconfirm group matching and also to explore possible relations between neural encoding and the development of written language skills (Table 1). Two scales from the Test of Word Reading (TOWRE) (single word reading measure [SWE], and phonemic decoding efficiency [nonword] reading measure [PDE]; Torgesen, Wagner, & Rashotte, 1999) were administered. The short form of the Wechsler Intelligence Scales for Children (WISC III, comprising the picture arrangement, block design, similarities and vocabulary subscales; Wechsler, 1992) was administered at the beginning of the longitudinal behavioural study. These four subscales of the WISC yield an estimate of full-scale IQ (pro-rated, see Sattler, 1982).

### 2.3 Experimental phonological task

A syllable stress perception task was selected to measure differences in phonological processing between children. Sensitivity to syllable stress was tested as part of earlier behavioural testing using an 80-trial same-different judgement task based on 4-syllable words (see Goswami, Mead, et al., 2013). The child heard the same word twice, with same or different stress (e.g., DIfficulty and diFFIculty; ‘different’ judgement required). Sensitivity to syllable stress (d’) was computed. Further task details can be found in Goswami, Mead, et al., 2013, Leong et al., 2011.

### 2.4 Noise-vocoded speech (NVS) paradigm

Participants were presented with 100 sentences twice, and asked to repeat them aloud. Each sentence comprised 4 words as 8-channel noise-vocoded speech. Participants were scored for the accuracy of word report (maximum score = 100 × 4 × 2 = 800). The sentences were semantically unpredictable but grammatically correct (e.g. *Arcs blew their cough*). Semantically unpredictable sentences were used to eliminate the use of compensatory contextual cues to identify the target words (Johnson et al., 2011). Participants received training with 4 sentences, each heard twice consecutively, first in the natural form and then in vocoded form. After each presentation the participant was asked to repeat the sentence. During the subsequent experimental test participants heard 100 pairs of processed sentences. The auditory stimuli were delivered binaurally through foam-tipped insert ear-phones (ER-1, Etymotic Research) at a comfortable hearing level. Sentences were again presented twice consecutively, however both presentations were in noise-vocoded form. Sentence pairs were presented in random order. After each presentation the participants were asked to repeat the sentence as best they could. Participants were given 7.5 s to respond. The number of words reported correctly was recorded for each child.

### 2.5 EEG preprocessing

EEG data were recorded and digitised with a 24-bit analog-to-digital (A/D) converter using the 129-channel EGI geodesic Sensor Net system. The sampling rate was 250 Hz. The data were lowpass filtered at 120 Hz prior to A/D conversion to prevent aliasing due to the presence of frequencies above the Nyquist frequency (half the sampling rate i.e. 250 Hz). Data pre-processing was done in MATLAB (MathWorks) using FieldTrip (http://fieldtrip.fcdonders.nl). The data were high pass filtered offline at 0.2 Hz and lowpass filtered at 80 Hz using 5th order high-pass^1^ and 6th order low-pass filters respectively. The EEG data were assessed for bad channels before and after epoching into responses to the individual sentences. Three channel measures were used to identify bad channels: low frequency power (<20 Hz), overall variance and mean correlation with all other channels. If any measure was 3 standard deviations from the mean of all channels that channel was identified as bad. Channels identified as bad in the continuous data or within single epochs were interpolated using the *ft_channelrepair* function in FieldTrip, which replaces a designated bad channel with the average of its neighbours weighted by distance. Bad trials were also removed, defined as trials where the individual trial variance was 3 standard deviations from the mean variance of all trials. Approximately 3.28% of trials were marked as bad, and the number of bad trials did not differ between groups (F(1, 44) = 2.861, *p* = 0.098, $\eta_{G}^{2}$ = 0.061). The individual trial variance was obtained by averaging the variance of all channels within a trial.

### 2.6 Stimulus reconstruction method

To inspect the strength of neural population encoding via EEG we employed reverse reconstruction (Bialek, Rieke, Van Steveninck, & Warland, 1991). This method has been used extensively to probe speech encoding in various contexts (Mesgarani et al., 2009, O’Sullivan et al., 2014). Here we employed it to assess stimulus encoding accuracy at the single sentence level in the three groups. A mapping from the resultant EEG to the presented stimulus envelope was estimated and this model was used to estimate the encoding accuracy of a novel single trial stimulus. To ensure that the model was not unfairly biased to the specific sentence or participant under test, each stimulus for each participant was reconstructed using a mapping obtained using data from the other 198 stimuli (as each sentence was presented twice) and from every *other participant* within that group. This was done for all 200 sentences. The encoding accuracy of each individual sentence was then estimated using a Pearson correlation between the reconstructed envelope and the actual stimulus envelope that was presented. The average reconstruction accuracy over all stimuli was then obtained for each participant. Envelope reconstruction accuracy was estimated in 5 AM frequency bands (0–2, 2–4, 4–6, 6–8, 8–10 Hz). To obtain the 0–2 Hz band we low-pass filtered at 2 Hz using a 6th order Butterworth filter and removed the DC component by subtracting the mean of the resultant envelope. The envelopes in the remaining four bands were obtained by low-pass filtering at the higher frequency and high-pass filtering at the lower frequency, again using 6th order Butterworth filters in all cases. Before carrying out the reconstruction analysis an anti-aliasing filter was applied to both the EEG and the stimulus envelopes (ft_resampledata.m) which were then downsampled to a sampling frequency of 25 Hz (to ease computational complexity).

Mappings were estimated based on a causal time window of 0–440 ms. Activity in the analysis window is potentially influenced by engagement with the task. In order to control for this attentional variable, an additional analysis in which performance was matched across groups was carried out. This identical analysis was restricted to a subset of the trials for which performance was matched between the groups. Trials in which participants reported 3 or 4 words out of 4 correctly were included in this analysis. This additional analysis controlled for potential encoding differences arising from differential engagement.

**Fig. 2:**
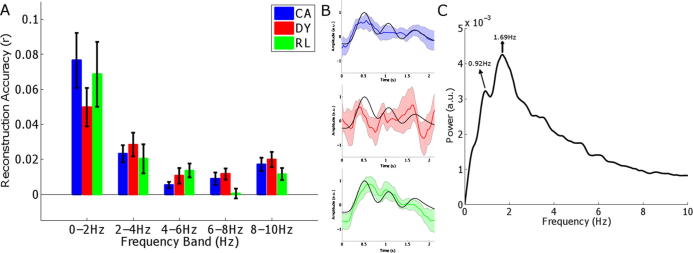
Stimulus reconstruction accuracy by group. Panel A shows the accuracy of reconstruction as assessed by the Pearson correlation between the actual stimulus envelope and the EEG reconstruction in each of the tested frequency bands. Black bars indicate the 95% confidence interval. Panel B shows group average envelope reconstructions in the 0–2 Hz band for a representative stimulus (*Roles teased his drain*) (coloured traces). The shaded coloured area shows the 95% confidence intervals of the reconstructions. The black trace indicates the actual 0–2 Hz envelope of the stimulus. Panel C shows the average frequency spectrum of the envelopes of the stimuli. Indicated are the modal frequency (1.69 Hz, which coincides with the syllable rate) and the prosody rate (0.92 Hz).

In all cases reconstruction accuracy for frequencies >4 Hz was low, albeit significantly above chance. This most likely reflects the fact that the average syllable rate in the natural speech stimuli was 1.7 syllables per second. The stimulus envelopes had a modal frequency of 1.69 Hz and another peak at 0.92 Hz, coinciding with the stress rate (see Fig. 2). Hence the two most prominent low-frequency speech rates (approximately equivalent to the linguistic stress and syllable rates) were in the 0–2 Hz band for our stimuli.

### 2.7 Beta power and delta-beta phase-amplitude cross-frequency coupling

Beta power was extracted from the subset of trials that were matched for performance (i.e. the same trials that were used for the sentence-matched stimulus reconstruction analysis). This was done using *ft_freqanalysis.m* function in fieldtrip. Power was obtained for each trial and individual subject averages were obtained. Power in the 18–22 Hz beta range was submitted to statistical testing. To obtain a measure of delta-beta phase-amplitude cross-frequency coupling the data were filtered into delta (0–2 Hz) and beta (18–22 Hz) bands. Delta activity was obtained by low-pass filtering at 2 Hz with a 6th order Butterworth filter. The DC component was removed by demeaning each trial. Beta activity was obtained by low-pass filtering at 22 Hz and high-pass filtering at 18 Hz. Both filters were 6th order Butterworth filters. All filtering was implemented using the *ft_preprocessing.m* function in fieldtrip. We extracted delta phase and beta amplitude by getting the angular component and absolute value of the Hilbert transforms of the delta-band and beta-band activity, respectively. Phase-amplitude coupling can be assessed by combining these signals into a single complex variable, z, as follows, $\text{z}[\text{n}]={\text{a}}_{\beta }[\text{n}]{\text{e}}^{\text{i}\varphi_{\delta }[\text{n}]}$, where n is the sample number, *a_β_* is beta amplitude and *φ_δ_* is delta phase (Canolty et al., 2006). Phase-amplitude coupling, also known as the modulation index, is obtained by getting the absolute value of the mean vector as follows, $M=|\frac{1}{N}\sum_{n=1}^{N}z[n]|$. For a totally random relationship between phase and amplitude, M = 0, and for perfect coupling, M = 1.

In order to investigate differential contributions of beta power and delta-beta cross-frequency coupling between the groups topographies were submitted to non-parametric permutation analyses at each electrode. We did this for all pairs of groups (i.e. CA vs. dyslexic, hereafter DYS, CA vs. RL and RL vs. DYS). This was implemented using the *ft_freqstatistics.m* function in fieldtrip. 1000 permutations were used to obtain the non-parametric statistics. For each permutation the data from the two groups under test were randomly partitioned and compared using a *t*-test. This results in an empirical distribution of t-values. The t-value of the original group difference was then compared to this distribution to assess statistical significance. Clusters of significant electrodes are then established by finding groups of statistically significant electrodes that neighbour each other. We controlled for multiple comparisons using non-parametric cluster based analysis (Maris & Oostenveld, 2007). For the cluster-based multiple comparison correction we obtained a cluster-level statistic by summing the test statistic over electrodes in each cluster. We then corrected the P-values by comparing the cluster-level statistics of the original data to the cluster-level statistics of all permutations.

## 3 Results

### 3.1 Behavioural tasks

As shown in Table 1, the children with dyslexia had average IQ but impaired reading and phonological awareness. The group difference in reading age was significant (F(2, 43) = 8.93, *p* = 0.000, $\eta_{G}^{2}=0.309$). The CA controls had a higher reading age than both the dyslexic group and the RL controls. A group effect of phonological awareness was also found (F(2, 43) = 4.38, *p* = 0.019, $\eta_{G}^{2}=0.169$). The CA controls showed greater sensitivity to syllable stress patterns than the dyslexic group. Accuracy of word report was also significantly different between the groups (F(2, 43) = 13.44, *p* = 0.000, $\eta_{G}^{2}$ = 0.385). The CA controls reported significantly more words accurately than the dyslexics (p = 0.002) and the younger RL children (p < 0.001). The latter two groups did not differ. Hence the children with dyslexia were performing at a similar level to children who were 2 years younger in their perception of noise-vocoded words and phonological awareness.

### 3.2 Low frequency envelope reconstruction accuracy

The group (DYS, CA, RL) by Frequency Band ANOVA (0–2, 2–4, 4–6, 6–8, 8–10 Hz) revealed a significant group × frequency band interaction (F(8, 172) = 3.840, *p* = 0.013, *ε* = 0.379, $\eta_{G}^{2}=0.103$). Post-hoc tests showed that stimulus encoding was significantly less accurate in the 0–2 Hz band for the dyslexic group compared to both the CA controls (*p* = 0.001, *dZ* *=* *−*0.38, Fig.2a) and the RL controls (*p* = 0.004, *dZ* *=* *−*0.35, Fig.2a). The CA and RL control groups did not differ in encoding accuracy in the 0–2 Hz band (*p* > 0.05, *dZ* = *−*0.1). Note that although the correlations shown in Fig.2a may appear low in magnitude, reconstruction accuracy was significantly greater than zero in the 0–2 Hz frequency band for all three groups (CA: t[22] = 9.28, *p* < 0.001, *d_Z_* = 1.94; DY: t[11] = 8.51, *p* < 0.001, *d_Z_* = 2.46; RL: t[10] = 6.89, *p* < 0.001, *d_Z_* = 2.08). There were no group differences for any other frequency band, although encoding accuracies were above chance in these bands also (and are of similar size to those found in adult speech perception studies, O’Sullivan et al., 2014). Thus the younger RL children were encoding the low-frequency speech envelopes as accurately as the older CA controls, even though they reported fewer words correctly. The dyslexic children both encoded this speech information significantly more poorly than both the CA and RL controls *and* reported fewer words correctly than the age-matched children. Fig.2b shows averaged group envelope reconstructions in the 0–2 Hz band for a representative stimulus. Envelope reconstruction accuracy is clearly atypical in the dyslexic group.

### 3.3 Low frequency envelope reconstruction accuracy, matched behavioural performance

Given that both DYS and RL behavioural performance was impaired compared to the CA controls, we ran a further analysis controlling for the potential confound of differential engagement with the task. Here we repeated the stimulus reconstruction analysis, but we only used trials where participants’ accuracy was 75% or higher. The analysis used the following number of trials: CA group, 120.83; DY group, 89.75; RL group, 86.55. Comparing behavioural performance for this subset of trials confirmed that accuracy of word report did not differ between the groups (*F*[2, 43] = 1.838, *p* = 0.171, $\eta_{G}^{2}=0.079$). Differences in encoding accuracy were then assessed using a second group × frequency (3 × 5) ANOVA. This again revealed a significant group x frequency interaction, (F(8, 172) = 38.329, *p* = 0.000, *ε* = 0.656, $\eta_{G}^{2}=0.474$). Post-hoc tests again showed a number of significant differences. CA controls had significantly more accurate envelope encoding than the DYS group (*p* < 0.001, *dZ* = −0.39) but equivalent accuracy to the RL group, *p* > 0.05, *dZ* = −0.06. RL controls had significantly more accurate envelope encoding than the DYS group (*p* = 0.017, *dZ* = −0.42). There were no group differences at any other frequency. Therefore differential engagement with the task does not explain the reduced encoding accuracy shown by the children with dyslexia. Rather, they have a basic speech encoding deficit compared to *both* CA and RL controls. Despite their envelope encoding deficit, they are presumably able to report the same number of words correctly as the younger RL children via their higher mental age (both groups scored in the normal range in the WISC, hence the mental age of the DYS group was around 14 years while the mental age of the RL group was around 12 years).

### 3.4 Relationship between low frequency encoding and phonological awareness

**Fig. 3:**
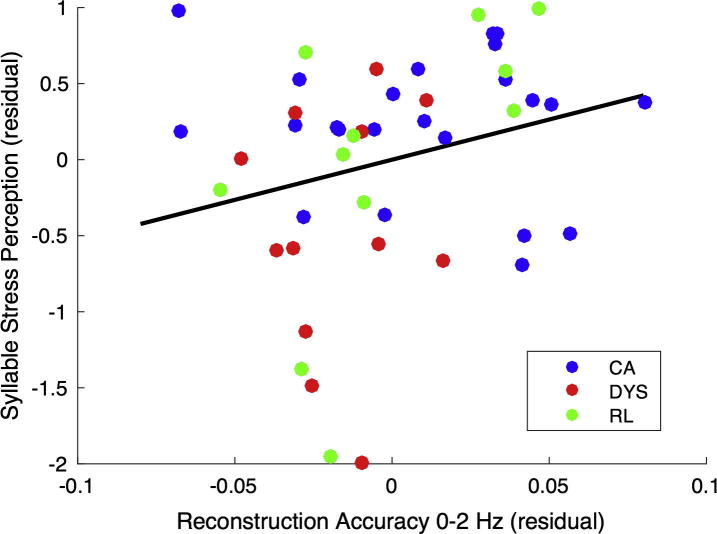
Partial correlation between phonological awareness and mean reconstruction accuracy, controlling for age and IQ. The figure shows the relationship (Spearman’s *ρ*) between mean reconstruction accuracy and performance on the lexical stress perception task. The scatter plot shows residual variables after removing the variability due to age and IQ using linear regression. The reconstruction accuracy measure is from the overall analysis, where performance on the word report task is not matched.

To assess whether individual differences in the reconstruction accuracy (0–2 Hz envelope) for each participant were related to phonological awareness (lexical stress perception), a partial correlation was computed using all trials. We controlled for age and nonverbal IQ measured at the time of the EEG testing (Block Design subscale of the WISC), and we used Spearman’s rho in order to minimise the potential influence of outliers. A significant relationship was found between reconstruction accuracy in the 0–2 Hz band and phonological awareness (lexical stress perception; *ρ* = 0.30, p < 0.05). The latter relationship is shown as Fig. 3, with the different groups identified in the scatterplot. Higher levels of reconstruction accuracy are found for the younger children, as also shown in Fig.2b, suggesting that the fidelity of bottom-up encoding may be greater early in development, when children have less exposure to the pragmatic and contextual aspects of language use. The more accurate the encoding of low-frequency envelope information, the better the child’s awareness of lexical stress.

### 3.5 Beta power and delta-beta phase-amplitude cross-frequency coupling

**Fig. 4:**
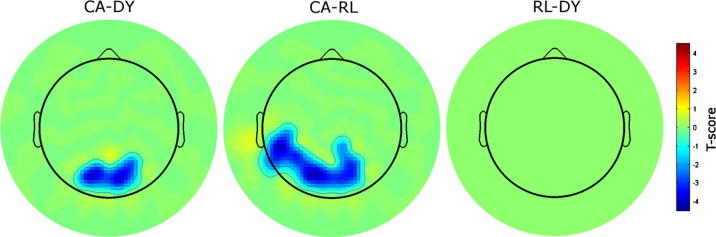
Group topography effects of beta (18–22Hz) power. Significant beta power differences at the topographical level between groups. Clusters that reached the significance threshold and survived correction for multiple comparisons are highlighted in blue (P_cluster-corrected_ < 0.05, based on non-parametric permutation analysis and cluster-based correction for multiple comparisons using 1000 random data partitions).

Finally, we investigated potential group differences in beta power and delta-beta phase-amplitude cross-frequency coupling. For beta power, topographical group differences were found between the CA and DYS groups and between the CA and RL groups (both P_cluster-corrected_ < 0.05), while no differences were found between the RL and DYS groups (P_uncorrected_ > 0.05 at all electrodes). For the CA vs. DYS comparison, the children with dyslexia showed significantly greater beta activity. This was confined to parietal areas (Fig. 4, left panel; the significant electrodes were PPO7, PO7, PP05, PPO3, PP04, PO4, O2, PPO6, PPO8 on the UI 10/5 system, see Jurcak, Tsuzuki, & Dan, 2007). For the CA vs RL comparison, the younger RL group also showed significantly greater beta activity than the CA group (Fig. 4, middle panel; the significant electrodes were CP3, Tp7, TTP7, P5, PPO7, P1, PP05, PPO3, Oz, PP04, PO4, CPP4, O2, PPO6, CPP2, PPO8). Hence in both comparisons, temporo-parietal electrode sites dominated the effects. The topographical distribution of the CA vs. RL difference effect was much more extensive than for the CA vs. DYS contrast, suggesting a role for maturation (as the RL children were 2 years younger). As well as a parietal effect, the CA vs. RL difference topographies showed greater activation for RL children in left motor and temporal/auditory areas compared to the older CA children. No significant differential effects were observed for the RL vs. DYS contrast. Regarding potential delta-beta phase-amplitude cross-frequency coupling differences between groups, no differences survived the correction for multiple comparisons (P_uncorrected_ > 0.05 at all electrodes).

## 4 Discussion

This study is the first to reveal that when listening to sentences, neural encoding of the low-frequency amplitude information in speech at the single sentence level is significantly worse in children with dyslexia compared to both CA-matched and RL-matched control children. Our developmental research design suggests that the observed deficit may be a fundamental one. The data showed significantly less accurate encoding of low-frequency envelope information in the 0–2 Hz frequency band by dyslexic participants in comparison to *both* typically-developing children of the same age who were matched for oral language experience (the CA match group), and younger children whose reading experience was matched to that of the children with dyslexia on standardised tests (the RL match group). These significant group differences in encoding accuracy remained robust when the data analysis was restricted only to sentences that were recognised correctly by all participants, ruling out differential engagement with the stimuli. Further, the accuracy of low-frequency envelope encoding was significantly related to individual differences in phonological awareness (lexical stress perception). These findings have critical implications for the aetiology of dyslexia and for remediation of the ‘phonological deficit’ in affected children.

### 4.1 Implications for aetiology and remediation

The primary locus of impairment in encoding speech information found here was within the EEG delta band (0–2 Hz). This is suggestive of linguistic impairments at the level of speech prosody and syllable parsing. Prosody is primarily carried in speech by slow amplitude modulations at temporal rates around 2 Hz (see Ghitza and Greenberg, 2009, Giraud and Poeppel, 2012). Accurate perception of speech prosody is fundamental to the development of a mental lexicon of word forms in infancy and childhood, across languages (Jusczyk et al., 1999, Mehler et al., 1988). Further, recent analyses of the amplitude modulation structure of Australian English infant-directed speech (IDS) show a modulation peak at 2 Hz, the “prosodic rate” (see Leong, Kalashnikova, Burnham, & Goswami, 2014), not at 4–6 Hz, the modulation peak found for adult-directed speech (ADS, see Greenberg, Carvey, Hitchcock, & Chang, 2003). These different modulation peaks imply that early in development, accurate encoding of low frequency envelopes (delta band) could play a crucial role in setting up a phonological lexicon (Leong & Goswami, 2015). Infants and young children who are relatively insensitive to low-frequency envelope information would benefit less from the prosodic information in IDS as they build their lexical phonological representations.

Indeed, a recent MEG study using a sentence listening task found that both adults and children with dyslexia showed impaired oscillatory entrainment to speech in the delta band, with reduced delta synchronisation originating in right primary auditory cortex (Molinaro et al., 2016). This appears to show that a delta-driven speech encoding difficulty does not ameliorate with development. Further, Molinaro et al., demonstrated impaired feedforward functional coupling in the dyslexics between the neuronal oscillations in the right hemisphere and those in left inferior frontal regions, areas involved in higher-order speech computation. If supported by future studies, these neural impairments would suggest that oral phonological remediation for developmental dyslexia should incorporate the prosodic phrase and the syllable in addition to the phoneme. As will be recalled, in the sentences used for this study the average syllable rate in the natural speech stimuli was 1.7 syllables per second, with the prosodic rate (the stressed syllable rate) occurring around once per second (the stimulus envelopes had a modal frequency of 1.69 Hz and another peak at 0.92 Hz). Hence our 0–2 Hz data may reflect impaired neural encoding at *both* the syllable and prosodic linguistic levels in children with dyslexia.

### 4.2 Anatomical origins of the deficit

It is known that low frequency speech envelopes are tracked in numerous anatomical regions including Heschl’s gyrus (HG), planum temporale (PT), superior temporal sulcus (STS), superior temporal gyrus (STG), the inferior parietal lobule (IPL) and inferior frontal gyrus (IFG, Zion Golumbic et al., 2013, Poeppel, 2014). These neuroanatomical data suggest that the group differences found in the current study most likely occur relatively early in neural processing, during auditory encoding. The current study used noise-vocoded speech as well as semantically unpredictable sentences. Both factors are likely to have reduced the neural involvement of higher linguistic areas such as angular gyrus (see also Golestani et al., 2013, Hartwigsen et al., 2014), and increased the engagement of lower-level areas such as superior temporal sulcus (STS, the first auditory area that performs speech-specific analysis, including for noise-vocoded stimuli, Overath, McDermott, Zarate, & Poeppel, 2015). Molinaro et al. (2016) reported that the brain regions whose delta band oscillations synchronised with speech in their participants were right and left auditory cortex, right and left STG, right middle temporal gyrus, and left IFG, comprising a feedforward network beginning in auditory cortex. Since the stimulus reconstruction method employed here establishes a mapping between the stimulus and the neural activity by estimating the *direct linear relationship* between the stimulus and the resultant EEG, the encoding accuracy estimates are biased towards measuring activity from lower-level auditory areas. Accordingly, although we cannot rule out contributions from IFG, on this basis we assume that primary auditory cortex, STS and STG are likely to be the regions most relevant to our extracted encoding accuracy measure.

### 4.3 The role of beta activity and temporal prediction

As noted earlier, recent work in neural speech processing has shown that delta-beta phase-amplitude coupling in auditory and motor regions underlies temporal prediction accuracy (Arnal et al., 2014). In the current study, no differences were found in delta-beta phase-amplitude coupling between the CA and DYS groups. There was also no difference in delta-beta phase-amplitude coupling between the RL and DYS groups. This suggests that impaired sensori-motor coupling was not associated with being dyslexic in this sample, and that impaired sensori-motor coupling and temporal prediction accuracy were not associated with being dyslexic in our task. The topographic data (Fig. 4) showed a significant difference between the CA and DYS children in observed beta power in parietal areas. Significant differences in beta activity between the CA and RL groups also occurred in parietal and frontal areas. However, as *both* the older reading-impaired children and the younger RL-matched children showed significant differences in beta activity in comparison to the typically-developing (CA-matched) children, the differential beta activity observed was most likely a correlate of task demands. One possibility is that our utilisation of noise-vocoded speech may have made greater demands than is typical during language processing on associated cognitive skills, such as working memory. In the current paradigm, however, there was no difference between the RL and DYS groups in beta activity, suggesting that differential beta activity is not a contributor to the envelope reconstruction findings. Indeed, mean beta power over frontal and parietal regions was not correlated to an earlier measure of phonological short-term memory administered to this sample of children (*ρ* = 0.06, *p* = 0.68). Finally, there is growing consensus that motor areas contribute to speech perception, particularly in difficult listening conditions (e.g. Davis and Johnsrude, 2003, Hervais-Adelman et al., 2012, Möttönen et al., 2014). Motor activity is classically considered to be in the beta band, hence is unlikely to contribute directly to the low frequency encoding deficit observed here, as only frequency bands spanning 0–10 Hz were considered. Furthermore, there was no observed difference between the CA and DYS groups in the beta band over motor areas, suggesting comparable motor activity between these two groups in this frequency range.

### 4.4 Theoretical implications for developmental dyslexia

The findings reported here are consistent with current neural computational accounts of speech encoding (Giraud and Poeppel, 2012, Gross et al., 2013) and with the temporal sampling theoretical framework for developmental dyslexia (Goswami, 2011). By these accounts, speech is encoded in part via the entrainment of neuronal oscillations in auditory cortex by amplitude modulations in the speech signal at multiple temporal rates simultaneously (delta, theta, beta, gamma; see Poeppel, 2014). Neural networks oscillating at these different temporal rates concurrently “sample” the speech signal in multiple temporal windows and encode its rich temporal characteristics (Luo and Poeppel, 2007, Poeppel, 2003). Temporal sampling theory proposed that atypical neural entrainment to the slower amplitude modulations in speech (<10 Hz) may contribute to the phonological processing impairments found in developmental dyslexia. Slower energy fluctuations (amplitude modulations <10 Hz) relate to speech prosody and rhythm, and difficulties in recovering prosodic and rhythmic structure from the speech signal would affect phonological development in children across languages. The neural data reported here for English, a stress-timed language (and by Molinaro et al., for Spanish, a syllable-timed language) suggest that similar developmental investigations in other languages could be important.

For example, impaired neural entrainment to low-frequency amplitude envelopes should affect the accurate representation of syllable stress and syllable boundaries (Goswami, 2011, Goswami, 2015), impairments that indeed characterise children with dyslexia in some languages (Goswami, Mead, et al., 2013 for English; Jiminez-Fernandez, Gutierrez-Palma, & Defior, 2014 for Spanish, see also Soroli, Szenkovits, & Ramus, 2010 for French [dyslexic adults]). Further, impairments in the auditory processing of amplitude envelope *rise times* (perceptual cues to modulation rates) are found in children with dyslexia in many languages (to date, Finnish, Spanish, Chinese, English, French, Dutch and Hungarian, Goswami, 2011, Goswami, 2015, for recent summaries). Research on oscillatory mechanisms shows that amplitude rise times phase-reset endogenous neuronal activity, functioning as “auditory edges” that enable alignment of neuronal rhythms with corresponding rhythms in speech, (Doelling et al., 2014, Gross et al., 2013). Cortical oscillations are organised hierarchically, with low frequency activity modulating processing at faster rates (Gross et al., 2013). This oscillatory hierarchy means that inaccurate or atypical low frequency envelope encoding in developmental dyslexia would also affect other levels of linguistic structure. For example, atypical entrainment related to linguistic prosodic structure would also affect the representation of syllables, phonological feet, onset-rimes and phonemes, which are encoded “downstream” by oscillations at higher (faster) temporal frequencies (see Goswami, 2015). These predicted downstream effects can be studied directly in future oscillatory work with dyslexic children (see Leong & Goswami, 2014, for a relevant study with dyslexic adults).

Indeed, the participants with dyslexia in the current study were drawn from a larger group of children whose reading and phonological development has been followed since they were aged 7 years (e.g., Goswami et al., 2011, Goswami, Huss, et al., 2013, Goswami, Mead, et al., 2013). The children with dyslexia have shown impaired phonological awareness and impaired auditory sensitivity to amplitude envelope rise times since the study began. Earlier testing of this cohort included an attention screen, using the Barkley scale (Barkley & Murphy, 1998); this inattention measure showed no association with the accuracy of encoding measure used here (*r* = 0.12, *p* = 0.56, for a subset of the current participants, 16 CA and all 12 dyslexics). Earlier EEG studies with these dyslexic children also revealed unimpaired ERPs for short rise times (15 ms) and impaired ERPs for longer rise times (90 ms) compared to CA controls (Stefanics et al., 2011). As rise times reflect amplitude modulation rates, this is consistent with impaired sensitivity to the amplitude modulation structure in speech at slower temporal rates.

Finally, it is worth noting that our data do not support the idea that the “phonological deficit” in developmental dyslexia arises from impaired access to *intact* neural representations (Boets et al., 2013, Ramus and Szenkovits, 2008). Rather, the data show that low frequency amplitude information in the 0–2 Hz band (delta band) in the speech signal is encoded significantly more poorly by children with dyslexia. Our envelope reconstruction data suggest that *prosodic* and *syllabic* information (both occurring in the delta band in our stimuli) are not encoded accurately when children with dyslexia are listening to speech. All languages vary syllable stress and prosodic phrasing, and therefore a low-frequency envelope encoding impairment could be expected to affect phonological representation (and consequently reading development) in all languages, even languages that do not use an alphabetic (phoneme-based) orthography. Nevertheless, our stimuli were both noise-vocoded and semantically unpredictable. Further research with natural, semantically predictable speech is required to assess the generalizability of the effects found here, ideally in a range of languages.

## Figures and Tables

**Table 1:** Participant Information showing age, IQ, reading, nonword reading, phonological awareness and performance on the noise-vocoded word report task.

	Groups			ANOVA		Post-hoc (Newman-Keuls)		
	CA (N = 23)	DY (N = 12)	RA (N = 11)	F(2, 43)	*P*	*p_CA vs. DY_*	*p_CA vs. RA_*	*p_DY vs. RA_*
Age (mths)	173.74 (12.58)	176.25 (16.31)	142.82 (9.03)	25.3	<0.001	>0.05	<0.001	<0.001
FSIQ (SS)^a^	111.96 (12.06)	113.33 (11.29)	102.91 (10.08)	2.69	=0.063	N/A	N/A	N/A
TOWRE word (SS)	100.87 (10.31)	86.92 (6.07)	101.36 (11.21)	9.45	<0.001	<0.001	>0.05	<0.001
TOWRE nonword (SS)^b^	102.48 (10.24)	79.75 (11.44)	99.18 (17.4)	13.3	<0.001	<0.001	>0.05	=0.002
Reading age (mths)	172.04 (23.28)	137.33 (27.91)	144.91 (23.83)	8.93	<0.001	=0.003	=0.01	>0.05
Syllable stress (d’)	4.53 (0.48)	3.86 (0.87)	3.94 (0.94)	4.38	=0.019	=0.05	=0.04	>0.05
Word report/800	540.96 (49.36)	462.5 (75.75)	439.36 (59.75)	13.44	<0.001	=0.001	<0.001	>0.05

*Note.* Standard deviations in parentheses.

a The scores in Table 1 are prorated full-scale IQ scores obtained at the outset of the longitudinal study.

b One RL-match child did not complete the TOWRE PDE scale (nonword reading) during the EEG session. As this is a longitudinal study, the standard scores from that child taken the previous year were used instead, and 12 months was added to the child’s previous year’s reading age.

## Footnotes

1 The order of the high-pass filter was automatically reduced by FieldTrip to fix filter instability.
